# *C-MYC, BCL2* and *BCL6* Translocation in B-cell Non-Hodgkin Lymphoma Cases

**DOI:** 10.7150/jca.36954

**Published:** 2020-01-01

**Authors:** Dayang Sharyati Datu Abdul Salam, Ei Ei Thit, Siew Hoon Teoh, Soo Yong Tan, Suat Cheng Peh, Shiau-Chuen Cheah

**Affiliations:** 1 Faculty of Medicine and Health Sciences, UCSI University, Kuala Lumpur, Malaysia.; 2 Advanced Molecular Pathology Laboratory, SingHealth Tissue Repository, Singapore.; 3 National University Hospital, Singapore.; 4 Sunway University Hospital, Selangor, Malaysia.

**Keywords:** B-cell Non-Hodgkin Lymphoma, *C-MYC*, * BCL2*, *BCL6*, gene abberation, FISH

## Abstract

*C-MYC*, *BCL2* and *BCL6* genes are the most commonly oncogenes involved in B-Cell lymphomas. Translocations of these oncogenes are associated with an aggressive clinical course. This study aims to elucidate the patterns of *BCL6*, *BCL2* and *C-MYC* gene aberrations among Malaysian B-cell Non-Hodgkin Lymphoma (NHL) using fluorescence *in situ* hybridization (FISH). Eighty-one B-cell NHL tissue blocks were retrieved between the year 2011 to 2015 and investigated using immunohistochemistry and interphase FISH dual colour break-apart probes of *BCL2*, *BCL6*, *C-MYC* and *IgH*. A significant difference was detected between the nodal and extranodal sites in all the *BCL2* (p=0.01), *C-MYC* (p=0.03) and *IgH* (p=0.006) cases except for *BCL6* (p=0.2). Our study showed that *BCL6* had the highest gene translocation while *BCL2/BCL6* had the most mixed aberrations of gain copies and translocation, however no mixed aberrations of gain copies and translocation was found in *C-MYC*. None of the mixed gain copies and translocation was found in any of the germinal centre B-cell (GCB) subtype of Diffuse Large B-cell Lymphoma, however, five were found in *BCL6* and *IgH* gene in the non-GCB subtype; while mixed gain copies and translocation cases of *BCL2* gene was found in the Follicular Lymphoma cases only. The study found interesting findings of *BCL2*, *C-MYC* and *IgH* gene aberrations between nodal and extranodal sites. This information might benefit future study in predicting prognosis and determine effective therapeutic strategies in the multi-ethnic populations of Malaysia as well as the Asian population.

## Introduction

There has been an intense upsurge worldwide for Non-Hodgkin lymphoma (NHL) incidence approximately 2-4% annually since the last seventy years 1. Based on the statistics presented, it was in accordance to the study done by Devesa and Fears 2 whereby western countries have the highest increased observed. Both males and females have seen an increased in all age groups however racial differences was observed in age-specific incidence curves up until the age of 45 and 35 for male and female respectively 3. The reasons for the increase include wider use of immunohistochemical method for screening and diagnosing cancer cell type, thus leads to lesser misdiagnosis cases of NHL as well as better classification of gray-zone lymphomas 4. In comparison to western countries, Asian countries have lower median age of NHL. The representative median age of patient in western countries was 68 years old 5. However, the median age of NHL patient in Asian countries such as Iran, Korea and Taiwan are between 50-55 years old 6-8. In the Global Cancer Facts 3^rd^ Edition 9, the global trend suggested that the increase of NHL cases in most developed countries reflects a true increase in disease occurrence. However, improvements in diagnostic methods and modification of classification may also play a role in the increase of NHL cases 10.

Double-hit translocation refer to translocation of *C-MYC* with another concurrent breakpoints such as *BCL2, BCL6, BCL1 or BCL3*. Triple-hit translocation consists of *C-MYC, BCL2* and *BCL6*. Currently, these gene translocations do not have any baseline characteristics aside from the genetic profile that can be confirmed through various genetic tests such as fluorescence *in situ* hybridisation (FISH). Also, morphologically they are difficult to distinguish and are usually unclassifiable B-cell lymphoma of intermediate characteristic between aggressive lymphoma, Diffuse Large B-cell Lymphoma (DLBCL), and very aggressive lymphoma, Burkitt Lymphoma (BL).

*C-MYC* translocation of t(8;14)(q24;q32) usually deregulate *C-MYC* expression. Rearrangement of the *C-MYC* gene situated on chromosome 8 next to the *IgH* gene, or lambda (λ) and kappa (κ) light chain genes subsequently caused upregulation of gene expression commonly observed in BL. In normal cells, *C-MYC* acts as a transcriptional regulator involved largely with cell cycle progression (from G1 to S phase) and the inhibition of terminal differentiation. Over-expression in normal cells sensitizes the cell to a variety of apoptotic triggers which leads the cell to resist cell death ultimately result in cancer*. C-MYC* gene rearrangement is reported to be rare occurrence in Follicular Lymphoma (FL). However, it was observed in 7-15% of *de novo* DLBCL 11 and 8% of post-transformation DLBCL 12. Several studies reported 13,14 the presence of *C-MYC* gene rearrangement upon transformation. Hence, it is highly probable that *C-MYC* plays a vital role in transformation of high grade B-cell malignancies.

*BCL2* translocation, t(14;18)(q32;q21), is observed in about 15-20% of DLBCL cases and approximately in 80-90% of FL cases 15. BCL2 came from the *BCL* family proteins which has a crucial role in cell apoptosis. BCL2 function as a pro-survival protein, represses apoptotic cell death, and protects cell from a wide range of cytotoxic insults including cytokine deprivation, ultraviolet irradiation. Deregulation of *BCL2* leads to over-expression of BCL2, making the cell to resist cell death. In contrast, *BCL2* inhibition eliminate cell survival advantage and allow apoptosis to occur. Similar to *C-MYC* translocation characteristic in DLBCL, chromosomal rearrangement that mix the *BCL2* gene situated on chromosome 18 involving the *IgH* gene deregulates the *BCL2* expression 16.

Coming from the same *BCL* family proteins, *BCL6* is essential for the development of germinal centre in B-cells. It acts as a transcriptional repressor in cell cycle control, proliferation and differentiation, apoptosis, and DNA damage response. Loss of normal control mechanisms regulating *BCL6* expression causes lymphoproliferative disease, resembling DLBCL. *BCL6* translocation, t(3q27), is responsible for up to 35% of DLBCL cases, the largest compared to other DLBCL gene translocations 17. Deregulation of *BCL6* located on chromosome 3 is believed to contribute to malignant transformation in germinal center-derived B cells (GCB). Detection of point mutations of the regulatory region of the *BCL6* gene have been frequently found in GCB and post-GCB lymphomas, including FL, DLBCL, and BL 18.

The genetic aberrations that are representative of specific subtypes of NHL mentioned previously can be identified by genetic diagnosis. Particularly, the use of FISH studies to diagnose NHL is becoming increasingly important. Interphase FISH is the most commonly applied technique for the demonstration of gene translocation, as it can be applied on paraffin embedded tissue, enabling studies on archival material. In comparison to immunohistochemical staining, interphase FISH are able to visualize gene expression pattern and can provide spatial and temporal information on understanding gene function which immunohistochemistry are not able to provide. Hence, the study aims to characterize the patterns of *BCL6, BCL2* and *C-MYC* gene aberrations in Malaysian B-cell NHL using interphase FISH.

## Patients and Methods

### Cases selection

A total of 81 B-cell Non-Hodgkin Lymphoma (NHL) were retrieved from a private hospital in Penisular Malaysia laboratory, the Pantai Premier Pathology Malaysia archives between the years 2011 to 2015. The demographic data of these patients, immunohistochemistry slides and tissue blocks were obtained from the Pantai Premier Pathology database by the referring clinician. All the B-cell NHL were sectioned at 3 µm for immunohistochemical staining using a panel of monoclonal and polyclonal antibodies according to Pantai Premier Pathology's standard operating procedure. For the DLBCL classification of GCB and non-GCB subtype, a panel of three antigens namely CD10, BCL6 and MUM1 was used according to Hans Criteria 19.

### Morphology

The materials were cut into 4 µm thick sections and stained with haematoxylin-eosin for histologic evaluation. All specimens were reviewed by a pathologist (PSC) for confirmation and classification according to the 2008 WHO Classification of tumours of haematopoietic and lymphoid tissues 4 using Leica DM500 light microscope (Leica Microsystems, Switzerland) graded reticule at x10 and x40 magnification.

### Interphase FISH and scoring

A total of 81 cases were examined using four interphase FISH dual colour break-apart probes namely *BCL2/18q21*, *BCL6/3q27, C-MYC/8q24* and *IgH/14q32* (Dako, Denmark). Haematoxylin-eosin stained tissue sections from representative formalin-fixed, paraffin-embedded tissue blocks were used to define the tumour areas. FISH analysis was performed on 2 µm tissue sections using FISH DNA break-apart probes and FISH Ancillary Kit (Vysis probe, Abbott, United States). The method was carried out at Advanced Molecular Pathology Laboratory (AMPL), SingHealth, Singapore according to AMPL's standard operating procedure. Briefly, the tissue slide was de-paraffinised and rehydrated prior to the procedure. After digestion with pepsin solution, FISH DNA Probe was added to the tissue sections and hybridized for three nights followed by stringent wash. Assessment of signals was performed using Zeiss Axioimager Fluorescent microscope (Germany) with the Metasystem's ISIS Metafer software (Germany) at x63 magnification. The cases performed depend on the availability of tissue block hence the total cases for each gene were different. The FISH analyses were divided into normal, translocated (>20% of the whole tumour population), gain copies (>50% of the whole tumour population) and both translocated and gain copies category.

### Statistics

Statistical analysis was completed using GraphPad Prism software (version 5.0; GraphPad Software Inc., San Diego, CA). χ^2^ or Fisher's exact tests were used for categorical parameters to identify significant relationships between variables. P value < 0.05 was considered statistically significant.

## Results

### Patients' Characteristics

The patients' demographic and clinical characteristics are summarized in Table 1. Among the 81 patients, there were 42 males (51.9%) and 39 females (48.1%) in this study. The male:female ratio is 1.08:1 and age ranged from 10-92 (median age of 55.8). The ethnic distribution were as follow: 23 Malays (28.4%), 42 Chinese (51.9%), 4 Indians (4.9%) and 12 Others consisted of Bumiputera and foreigners with undetermined country of origin (14.8%). There were 57 DLBCL cases (70%), 15 FL cases (19%), 1 BL case (1%), 1 DLBCL-BL like case (1%), 4 Marginal Zone Lymphoma (MZL) case (5%), 1 Mucosa-Associated Lymphoid Tissue (MALT) cases (1%), 1 Mediastinal B-cell Lymphoma case (1%) and 1 DLBCL-Plasmablastic Lymphoma case (1%). In terms of lymphoma sites, B-cell lymphoma originating from nodal sites was 42 (52%) and extranodal sites was 39 (48%). The most common extranodal sites are gastrointestinal (12 cases, 31%) and Waldeyer's ring (8 cases, 21%) and 19 others for example in the brain or spleen.

### Prevalence of *BCL2 / BCL6 / C-MYC / IgH* gene aberrations

A summary of all the FISH prevalence are given in Table 2. Out of the 81 cases, only 73 cases had successful *BCL2* FISH assay, 67 patients had successful *BCL6* FISH assay, 72 successful FISH assay were collected for *C-MYC* and 64 cases had successful *IgH* FISH assay. In DLBCL cases, *BCL6* and *IgH* gene aberrations recorded the highest with 19 and 20 cases respectively. In terms of DLBCL cell-of-origin (COO) classification, there was no significant difference between the gene aberrations to GCB and non-GCB type except for *BCL6* (*p*=0.03*). *BCL2* and *IgH* were the highest gene aberrations in FL cases with 6 and 7 cases respectively. One *BCL2* gene aberrations was found in MZL and one *C-MYC* and *IgH* gene aberrations were found in a BL case. Although female had slightly higher *C-MYC* and *IgH* gene aberrations compared to male, there was no significant difference found between them. While, Chinese had the highest gene aberrations in all the FISH *BCL2, BCL6, C-MYC* and *IgH* assays although there was no significant difference found among the Malaysian ethnics. Significant difference was detected between the nodal and extranodal sites in all the *BCL2* (*p*=0.01**), *C-MYC* (*p*=0.03*) and *IgH* (*p*=0.006**) except for *BCL6* (*p*=0.2), although higher *BCL6* gene aberrations were found in the nodal compared to extranodal sites.

### Analyses of *BCL2 / BCL6 / C-MYC* and *IgH* gene aberrations

Table 3 summarised the FISH analysis of *BCL2, BCL6, C-MYC* and *IgH* gene aberrations according to NHL subtypes. Among the 57 DLBCL cases, there were 13 *BCL2* gain (22.8%) and 3 (5.3%) *BCL2* translocated cases. While, FL had 4 (26.7%) *BCL2* translocated cases and 2 (13.3%) *BCL2* translocated and gain cases. In addition, 1 *BCL2* gain case was found in MZL. On the other hand, there were 6 *BCL6* gain (10.5%), 10 (17.5%) *BCL6* translocated cases and 3 (5.3%) *BCL6* translocated and gain. While, FL had 3 (20%) *BCL6* translocated cases and 1 (6.7%) *BCL6* gain case. There was no *BCL6* gene abnormalities found in Other NHL subtypes. In the 57 DLBCL cases for *C-MYC* aberrations, there were 5 (8.8%) *C-MYC* gain and 4 (7.0%) *C-MYC* translocated cases. While, FL had only 2 (13.3%) *C-MYC* gain cases. In addition, 1 *C-MYC* translocated case was found in BL. While for the *IgH* gene abnormalities, among the 57 DLBCL cases, there were 1 (1.8%) *IgH* gain, 17 (29.8%) *IgH* translocated and 2 (3.5%) *IgH* translocated and gain cases. Apart from that, FL had only 7 (26.7%) *IgH* translocated cases.

### Double and triple aberrations among *BCL2, BCL6* and *C-MYC*

There were 16 cases found to have concurrent genetic aberrations through FISH analyses on *BCL2, BCL6* and *C-MYC*. These cases were investigated further by doing comparison of their immunephenotype and genetic features as shown in Table 4 while summary of the gene aberrations are shown in Table 5. Of the 16 cases, 13 were DLBCL and 3 were FL cases. There was equal proportion of male and female however higher aberrations were found in the nodal sites (12 cases) than extranodal sites (4 cases).

**Figure 1:**
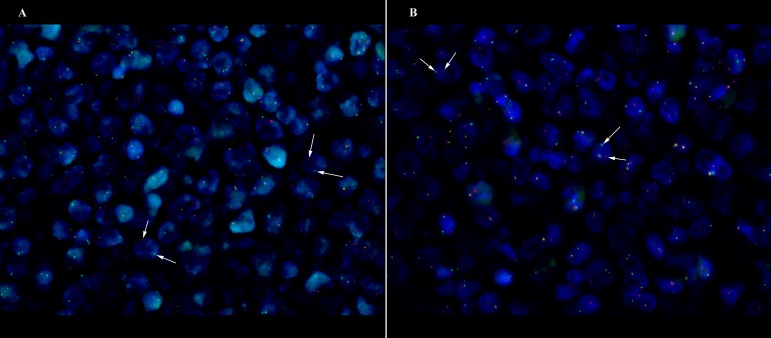
** DLBCL case of GCB origin with double-hit *C-MYC/BCL6* translocation (Case 6) at x63 magnification.** White arrow shows the discrete red and green signals. (A) *C-MYC* translocation (B) *BCL6* translocation.

One double-hit lymphoma of *BCL6/C-MYC* was established in DLBCL Case 6 (Fig 1), however both of the BCL6/C-MYC protein expression showed less than 50% and 40% respectively with Ki67 of 40%. Apart from that, two translocation of *BCL2/BCL6* (Case 3 and Case 14) cases were found with the Case 14 having additional 3-4 gain copies of *C-MYC*. The corresponding BCL2/BCL6 co-expression show positive expression (Case 3) and more than cut-off values (Case 14). There were three cases (Case 7, 12 and 15) showing triple gene aberrations of *BCL2/BCL6/C-MYC* with at least 3 gain copies of chromosome or trisomy 3. The three cases corresponding protein expression presented more than 70% BCL2, more than 40% C-MYC and all were non-GCB subtypes.

Comparing the genetic aberrations with the protein expression of the 16 cases showed that all of the 16 cases had positive protein expression for both BCL2 and BCL6. In the 13 cases of DLBCL, all of them had BCL2 protein expression of more than 70%. 9 DLBCL cases had BCL6 protein expression of more than 50% and only 6 DLBCL cases had more than 40% C-MYC protein expression. In the FL cases, 2 had *BCL2/C-MYC* gene aberrations and the other had *BCL2/BCL6* translocation. In the cases involving extranodal sites, all 4 cases had gain chromosomes of at least 3 copies in *BCL2. BCL2/BCL6* had the highest gene aberrations combination (6 cases) compared to *BCL2/C-MYC* (4 cases) or *BCL6/C-MYC* (1 case). With the exception of Case 1, 6 and 15, the cell proliferation for the 16 cases showed at least 50% Ki67 index.

## Discussion

In the present study, we reported the prevalence and genetic abnormalities of 81 cases of B-cell NHL among Malaysian patients using interphase FISH. Our findings revealed there was a significant difference detected between the nodal and extranodal sites in *BCL2, C-MYC* and *IgH*. The findings coincide with a study 20 investigating on metaphase comparative genomic hybridization (CGH) and their results showed nodal sites having more frequent amplifications than extranodal particularly of *BCL2*. The differences could readily be explained by the alterations in the behaviour of the cell types from the sites they develop 21. The findings connected previous evidences 22,23 that high gene aberrations at site-specific lymphomas may increase the number of lymphocytes, hence higher chances of a malignant clone developing. In addition, certain local factors such as exposure to numerous types of antigens and activation of the B-cell receptor (BcR) signalling pathway are known to increase the risk of lymphomas at nodal or extranodal sites 24. This is important as extranodal lymphoma is distinctive from nodal in numerous ways, such as in treatment strategies and prognosis. Apart from that, it is shown that among Asian population, extranodal sites such as gastrointestinal tract, nasal cavity and tonsils are the commonest sites and have high incidence compared to nodal sites 25. Undoubtedly, there is more to be learned of DLBCL pathogenesis at distinct extranodal sites since there is also emerging cases of differences and similarities in somatic genetic abnormalities particularly between DLBCL arising at different sites 26,27. However, there is little evidence in these data to suggest that aberrations in the nodal and extranodal sites could have effects on the clinical course and survival due to insufficient information of the patients.

*BCL2* translocation was found in 40% and 32% of our 13 FL and 51 DLBCL cases respectively. The *BCL2* translocation is the cytogenetic hallmark for approximately 90% of FL cases. Although the incidence of FL among Asian is considerably smaller compared to Caucasian, a Japanese study 28 found high (81%) *BCL2* translocated FL cases using FISH analysis in contrast to our FL findings with only 40% *BCL2* translocation. Similar findings in the Western countries, Germany 29 generated quite similar results with only 53% of *BCL2* translocation in early stage FL cases. Apart from the reason that we had considerably low FL sample size, it was also suggested that the discrepancy between the findings could be due to technique differences as suggested by a study 30 that investigated variations of the *BCL2* translocation incidence in FL. Aster and Longtine 30 examined data using methods that eliminate most false-negative results by detecting *BCL2* sequence rearrangements outside of the MBR (major breakpoints) and mcr (minor breakpoints) sites. In addition, *BCL2* translocation is not sufficient to cause B-cells transformation as seen in *BCL2* transgenic mice, which only develop lymphomas after secondary chromosome modifications and long latency period 31. Marin *et al.* 32 and Tanaka *et al.* 33 have both established *in vivo* that *BCL2* deregulation caused by translocation of *BCL2* increases cell survival by preventing apoptosis, prompting the cell to acquire secondary chromosomal aberrations. *BCL2* gene aberrations, other than t(14;18)(q32;q21), such as 18q21 amplification or activation of the nuclear factor-kB pathway, has been suggested to be the main responsible act for dysregulation of BCL2 protein expression in the DLBCL non-GCB subtype 34.

It was also found that in our FISH analysis of 50 DLBCL cases, the frequency of *C-MYC* aberrations of 18% was quite similar to what have been reported in Western populations 35. *C-MYC* rearrangement at band 8q24, is associated with a poor prognosis and reported in approximately 15% of DLBCL cases 35. However, supported data to incriminate geographical or genetical factors between DLBCL in South East Asian and in Western countries needs further observation that can be confirmed in larger studies. Deregulation of *C-MYC* has been implicated with aggressive lymphomas and adverse prognosis in B-cell malignancies as it plays a role by promoting cell cyle progression and tumour proliferation in DLBCL. Using the same samples, we had identified the MYC involvement in STAT3-mediated pathway in EBV (+)-DLBCL cases 36. Substantial evidence 37 of both *in vitro* and *in vivo* models have provided that *MYC* prompts many genes that involved in ribosome biogenesis including genes associated in glucose and glutamine metabolism to accommodate the growing lymphoma cells. Translocation of *MYC* has been described as the genetic hallmark and the driving oncogene for BL 38 as presented in our only BL case. BL is a germinal center tumour harbouring somatic hypermutation of the *Ig* genes and is characterized by the presence *MYC* and *IgH* translocation. Although, *C-MYC* is the defining feature to BL, it has also been recognized in other NHL B-cell lymphomas such as DLBCL and FL whereby MYC overexpression has been associated in the transformation of FL 39.

On the other hand, there was no significant difference for *BCL6* gene aberrations found between the nodal and extranodal sites. *BCL6* translocation and clinical features of B-cell NHL particularly DLBCL has been the subject of controversy. Several studies 40,41 reported that *BCL6* gene translocations occured frequently in extranodal than in nodal sites. Subsequent studies by Offit *et al.* 42 found that extranodal DLBCL patients had more frequent gene rearrangements than those found in the nodal sites of DLBCL. However, contradicting report 43 which also supported findings in this study found that there was no difference in the *BCL6* translocation between the DLBCL sites.

Our DLBCL findings also revealed that *BCL6* had the highest gene translocation (40%) compared to *BCL2* and *C-MYC*. The incidence of *BCL6* translocation in our study supported the evidence that *BCL6* gene translocation in B-Cell NHL constituted about 30-40% of all DLBCL 44,45. In addition, 6% of our FL cases also had BCL6 gene aberrations. Although it is unusual, previous study showed that 5-15% of FL patients had *BCL6* rearrangement that occurred in the both low-grade and high-grade FL 29, 46. It was suggested that FL cases with translocation other than *BCL2* may have unconventional molecular mechanisms or at least an alternate initiating event of FL 29. About 13 out of 23 cases of the *BCL6* gene aberrations in this study involved *IgH* gene while the rest are with *BCL2* and *C-MYC*, showing the diversity of *BCL6* translocation partner as mentioned by Cattoretti *et al.* 47. It is identified that non-*IgH/BCL6* translocated cases are under the effect of the regulatory regions in *BCL6* and some of these partner genes expression have role(s) in the development of DLBCL 48,49. *BCL6* gene rearrangement has been associated with poor prognosis when a study 50 evaluating *BCL6* translocation using FISH analysis found that translocation of *BCL6* in DLBCL patients had significantly lower overall survival than those without translocation. Consistent to what has been mentioned previously 51, we found significant higher *BCL6* gene abnormalities in non-GCB subtype (38%) than in GCB subtype (2%) (p=0.03), suggesting that the DLBCL GCB and non-GCB subtype have separate molecular mechanisms of DLBCL pathogenesis responsible for the abnormal protein expression. However, *BCL6* gene aberrations were not correlated with high BCL6 expression level in our study (p=1.00). Akyurek *et al.* 52 reported that there was no correlation between *BCL6* rearrangement with the level of BCL6 protein expression. However, this discrepancy can also arise due to the differences in the staining and scoring methodologies, cut-off values, and sample populations.

There were also 8 cases with *IgH* only gene aberrations which warrant further investigations. It is known that *IgH* is the first rearranged gene in B-cell development and an early marker of clonal B lymphoid processes, with light chain genes for κ and λ chains occurring only after heavy chain gene rearrangement has occurred through allelic exclusion 53. Under normal circumstances, only one of the two *IgH* alleles is functional due to its monoallelical nature and to facilitate allelic exclusion, the other allele is non-functional for several reasons, in this particular case the non-functional allele is translocated but encoded on a dysfunctional Ig chain therefore cannot be assembled into a surface-expressed BcR or an antibody 54. Another reason *IgH*-specific gene aberrations could occur was due to the problematic nature of the FISH *IgH* probe whereby we used three times signal diameter for *IgH* image analysis confirmation of translocation in our study. Due to the unique mechanism of V(D)J recombination, the green signals will be weak due to deletion. Sometimes following a translocation, a signal may be lost as the derivative partner chromosome is deleted or if the probe has been deleted by recombination. Hence, *IgH* green probe may faintly cross hybridise with small regions located on 15q11.2 and 16p11.2 showing tiny green dots accompanying the larger *IgH* green signals.

With regards to double or triple aberrations among the three genes, our study had one double-hit lymphoma of *BCL6/C-MYC* found in DLBCL Case 6, with both of the BCL6/C-MYC protein expression showing less than 50% and 40% respectively and Ki67 of 40%. The BCL6 and C-MYC cut-off values were carefully considered and adopted from previous studies 55,56 investigating the C-MYC, BCL2 and BCL6 expression in DLBCL patients. In addition, our study also found two cases harbouring both *BCL2/BCL6* translocation at the nodal site. One of this *BCL2/BCL6* double translocation were discovered in low grade FL case which was in accordance with previous studies 57-59 that reported the *BCL2/BCL6* translocation was predominantly low grade FL. Although there are contrasting evidence with regards to *BCL2* and *BCL6* rearrangement prognostic impact 50, consensus exist that *C-MYC* rearrangement is the worse prognostic marker for DLBCL patients treated with either standard CHOP or R-CHOP^5^.

Pfreundschuh 60 mentioned that, apart from translocation, FISH failed to detect other gene aberrations; however our study found other types of *C-MYC, BCL2* and *BCL6* gene aberrations such as gain copies which was detected through FISH break-apart probe analyses. There were three DLBCL cases having at least trisomy 3 of *BCL2/BCL6/C-MYC* and all were non-GCB subtypes. Asian and Western 61 studies revealed that the ABC subtype of DLBCL has frequent trisomy 3 especially involving chromosome 3 (*BCL6*) and 18 (*BCL2*) as defined by gene expression profiling. On the other hand, the small population of the GCB subtype in this study was found to be less compared to the non-GCB subtype, consistent with previous studies 62 on the prevalence of GCB-subtype on Asian patients. It was found that Asian DLBCL patients had less frequent GCB subtype compared to Caucasian patients, a difference that may be attributed to ethnic factors.

In summary, few interesting patterns emerged that should be interpreted with caution. Our data suggest that there is a significant difference between the sites of lymphoma hence suggest that lymphoma sites played an important role for gene abnormalities for *BCL2, C-MYC* and *IgH*. In support with other previous studies, *BCL6* remained the highest gene aberrations for B-cell NHL and *BCL2/BCL6* gene aberrations frequently occurred compared to other gene aberrations combinations. Since this is a retrospective study and we do not correlate our results with survival data of the patients, we are not able to establish prognostic implications particularly of a nodal genetic signature. However, our report on the incidence and pattern of *BCL6*, *BCL2* and *C-MYC* genes can serve as a platform for future studies to predict prognosis and determine therapeutic strategies in the multi-ethnic population of Malaysia as well as the diverse Asian population.

## Figures and Tables

**Table 1:** Demographic and clinical characteristic of 81 patients with B-Cell NHL

	N (%)
**Gender**	
Male	42 (52)
Female	39 (48)
**Age**	
<60 years	48 (59)
≥60 years	33 (41)
**Ethnic**	
Malay	23 (28)
Chinese	42 (52)
Indian	4 (5)
Others	12 (15)
**Sites**	
Nodal	42 (52)
Extranodal	39 (48)

**Table 2:** Prevalence of *BCL2 / BCL6 / C-MYC* / *IgH* gene aberrations in B-Cell NHL patients

	*BCL2* (n= 73)			*BCL6* (n= 67)			*C-MYC* (n= 72)			*IgH* (n= 64)	
	Normal N (%)	Aberrations N (%)		Normal N (%)	Aberrations N (%)		Normal N (%)	Aberrations N (%)		Normal N (%)	Aberrations N (%)
**B-cell NHL**	*p* = 0.22			*p* = 0.06			*p* = 0.87			*p* = 0.06	
DLBCL	35 (48)	16 (22)		28 (42)	19 (28)		41 (57)	9 (13)		24 (38)	20 (31)
FL	7 (10)	6 (8)		7 (10)	4 (6)		11 (15)	2 (3)		4 (6)	7 (11)
Others	8 (11)	1 (1)		9 (13)	0 (0)		8 (11)	1 (1)		8 (13)	1 (2)
**Gender**	*p* = 1.0			*p* = 0.8			*p* = 0.54			*p* = 0.46	
Male	25 (34)	12 (16)		21 (31)	12 (18)		32 (44)	5 (7)		19 (30)	12 (19)
Female	25 (34)	11 (15)		23 (34)	11 (16)		28 (39)	7 (10)		17 (27)	16 (25)
**DLBCL COO**	*p* = 0.47 (n=51)			*p* = 0.03* (n=47)			*p* = 0.43 (n=50)			*p* = 0.73 (n=44)	
GCB	9 (18)	2 (4)		10 (21)	1 (2)		11 (22)	1 (2)		6 (14)	4 (9)
Non-GCB	26 (51)	14 (28)		18 (38)	18 (38)		30 (60)	8 (16)		18 (41)	16 (36)
**Site**	*p* = 0.01*			*p* = 0.2			*p* = 0.03*			*p* = 0.006*	
Nodal	21 (29)	17 (23)		21 (31)	15 (22)		29 (40)	10 (14)		14 (22)	21 (33)
Extranodal	29 (40)	6 (8)		23 (34)	8 (12)		31 (43)	2 (3)		22 (34)	7 (11)
**Ethnic**	*p* = 0.54			*p* = 0.78			*p* = 0.49			*p* = 0.17	
Malay	12 (16)	7 (10)		13 (19)	6 (9)		15 (21)	4 (6)		13 (20)	5 (8)
Chinese	26 (36)	13 (18)		21 (31)	13 (19)		31 (43)	7 (10)		18 (28)	15 (23)
Others	12 (16)	3 (4)		10 (15)	4 (6)		14 (19)	1 (1)		5 (8)	8 (13)

COO, cell-of-origin

**Table 3:** FISH analysis of *BCL2, BCL6, C-MYC* and *IgH* gene aberrations

	DLBCL, n=57N (%)	FL, n=15 N (%)	Others, n=9 N (%)
**BCL-2**			
Gain	13 (22.8%)	0	1 (11.1%) *MZL
Translocated	3 (5.3%)	4 (26.7%)	0
Translocated & Gain	0	2 (13.3%)	0
**BCL-6**			
Gain	6 (10.5%)	1 (6.7%)	0
Translocated	10 (17.5%)	3 (20.0%)	0
Translocated & Gain	3 (5.3%)	0	0
**C-MYC**			
Gain	5 (8.8%)	2 (13.3%)	0
Translocated	4 (7.0%)	0	1 (11.1%) *BL
Translocated & Gain	0	0	0
**IgH**			
Gain	1 (1.8%)	0 (0.0%)	0
Translocated	17 (29.8%)	7 (46.7%)	1 (11.1%) *BL
Translocated & Gain	2 (3.5%)	0	0

MZL, Marginal Zone Lymphoma; BL, Burkitt Lymphoma

**Table 4:** Immunohistochemical and genetic features of the 16 DLBCL and FL cases

No.	Diagnosis	*BCL2*		*BCL6*		*C-MYC*		Ki67	*IgH*
		IHC	FISH	IHC	FISH	IHC	FISH	IHC	FISH
Case 1	FL	positive	T&G	positive	x	x	G (3- 4 cp)	x	x
Case 2	FL	positive	T	positive	N	x	G (up to 3 cp)	>80	T
Case 3	FL	positive	T	positive	T	x	N	50	T
Case 4	DLBCL	>70%	G (up to 3 cp)	<50%	G (3- 4 cp)	<40%	N	>90	x
Case 5	DLBCL	>70%	G (2- 3 cp)	<50%	T	<40%	N	>90	N
Case 6	DLBCL	>70%	N	<50%	T	<40%	T	40	T
Case 7	DLBCL	>70%	G (3- 4 cp)	>50%	G (3- 4 cp)	>40%	G (3- 4 cp)	50	G (3- 4 cp)
Case 8	DLBCL	>70%	G (up to 3 cp)	~50%	x	<40%	T	>80	x
Case 9	DLBCL	>70%	G (2- 3 cp)	>50%	N	>40%	T	~50	N
Case 10	DLBCL	>70%	G (3- 4 cp)	~50%	T&G	<40%	N	>95	N
Case 11	DLBCL	>70%	G (3- 4 cp)	>50%	G (2- 3 cp)	>40%	N	90	N
Case 12	DLBCL	>70%	G (4- 5 cp)	>50%	G (up to 3 cp)	>40%	G (up to 3 cp)	~100	N
Case 13	DLBCL	>70%	G (4- 5 cp)	>50%	T&G	>40%	G (3- 4 cp)	~100	T&G
Case 14	DLBCL	>70%	T	>50%	T	<40%	G (3- 4 cp)	>90	T
Case 15	DLBCL	~70%	G (5- 6 cp)	<50%	G (up to 3 cp)	>40%	G (3- 4 cp)	x	T&G
Case 16	DLBCL	>70%	G (3- 5 cp)	~50%	T&G	<40%	N	80	N

N, Normal; G, Gain copies; T, Translocation; T&G, Translocation & Gain copies; cp, copies; X, Missing/No tissue left

**Table 5:** Summarise of gene abnormalities among *BCL2 / BCL6 / C-MYC / IgH* in 16 cases of B-cell NHL

	N
	**Male (n=8), Female (n=8)**
	**Nodal (n=12), Extranodal (n=4)**
***BCL2/BCL6***	
*BCL2/BCL6* - (G)	2
*BCL2/BCL6* - (T)	1
*BCL2* (G) / *BCL6* (T)	1
*BCL2* (G) / *BCL6* (T&G)	2
***BCL2/C-MYC***	
*BCL2* (G) / *C-MYC* (T)	2
*BCL2* (T) / *C-MYC* (G)	1
*BCL2* (T&G) / *C-MYC* (G)	1
***BCL6/C-MYC***	
*BCL6/C-MYC* - (T)	1
***BCL2/ BCL6/C-MYC***	
*BCL2/ BCL6/C-MYC* - (G)	3
*BCL2/C-MYC* (G) & *BCL6* (T&G)	1
*BCL2/ BCL6* (T) & *C-MYC* (G)	1

G, Gain copies; T, Translocation; R&G, Translocation & Gain copies
